# Probiotics in Periodontal and Peri-Implant Health Management: Biofilm Control, Dysbiosis Reversal, and Host Modulation

**DOI:** 10.3390/microorganisms10112289

**Published:** 2022-11-18

**Authors:** Massimo Amato, Federica Di Spirito, Francesco D’Ambrosio, Giovanni Boccia, Giuseppina Moccia, Francesco De Caro

**Affiliations:** Department of Medicine, Surgery and Dentistry, University of Salerno, 84084 Salerno, Italy

**Keywords:** probiotic, probiotics, dysbiosis, host modulation, periodontal, periodontitis, periodontal disease, peri-implantitis, peri-implant disease, orthodontics, orthodontic treatment

## Abstract

Periodontitis and peri-implantitis are microbially associated diseases of the tissues supporting the teeth and dental implants that are mediated by host inflammation and eventually lead to tooth and dental implant loss. Given the probiotics’ role in biofilm control, dysbiosis reversal, and host modulation, their potential beneficial effects on the improvement of periodontitis and peri-implantitis have been recently investigated. Moreover, probiotics use has also been proposed in periodontal health management in patients undergoing fixed orthodontic therapy. Therefore, the present study aimed to review, considering the periodontal microbiome composition around teeth and dental implants in healthy and pathological conditions, the putative favorable effects of probiotics on gingivitis, periodontitis, and peri-implantitis. The secondary aim of the present narrative review was to synthesize the supporting evidence and proposed protocols for probiotics use as adjuncts in periodontitis and peri-implantitis treatment and the periodontal health management of orthodontic patients with fixed appliances. Contrasting findings from the literature may be due to the different methods, posology, and duration of probiotics prescriptions and due to the heterogeneous biological and clinical measurement methods employed. Thus, no definitive conclusions could be drawn about the effectiveness of probiotics in periodontal management, both in healthy and pathological conditions. Further studies are needed to validate probiotics for periodontal management and provide recommended protocols.

## 1. Introduction

The oral cavity is an ideal habitat for the growth of numerous species of microbiota that establish a delicate balance between them. In detail, healthy subgingival biofilm is a microenvironment that includes both Gram-positive bacteria and some Gram-negative species that co-aggregate into communities [1]. Conversely, in dysbiotic conditions, such as periodontitis and peri-implantitis, this balance between Gram-positive and Gram-negative changes in favor of some species [1]. 

Periodontitis is an inflammatory disease of the supporting tissues of the teeth, which leads to the loss of bone and periodontal ligament, up to the loss of the teeth [2,3]. Periodontitis etiology comprises the presence of microorganisms, and its pathogenesis is known to rely on the host-mediated immune-inflammatory response, although the interplay between the oral microbiome, host response, and periodontitis development is not completely understood [4,5,6]. The inflammation of the periodontal tissues, together with the dysbiotic phenomena of the periodontal microbiome, would also seem to be involved in the pathogenesis of several systemic conditions and inflammatory, degenerative, and neoplastic disorders, influencing, in turn, periodontitis onset and progression [7,8]. Periodontitis progression rate is assessed, directly, through the clinical and radiographic evidence of periodontal destruction assessed over time and, indirectly, through biofilm accumulation [9,10,11,12,13]. 

Based on the definitions stated in the 2017 World Workshop on the classification of periodontal and peri-implant diseases and conditions, peri-implantitis, which occurs in the tissues around dental implants, is characterized by bleeding on probing, increased probing depth, and progressive bone loss, and has an etiopathogenesis similar to that of periodontitis [2,14,15,16,17].

Common treatments for periodontitis and peri-implantitis include nonsurgical and surgical management, mainly aimed at mechanical debridement and occasionally coupled with antibiotics [18,19]. More recently, some authors have also suggested the use of probiotics as adjuvants in periodontitis and peri-implantitis therapy [4,20,21].

Probiotics are living microorganisms that, given in certain quantities in food or as food supplements, confer health benefits on the host [22,23]. In particular, in vitro and animal evidence have highlighted that probiotic preparations composed of dead cells and their metabolites can also exert a biological response in the host. Indeed, probiotics have been first used for the treatment of vaginal and intestinal infective and inflammatory conditions. 

Subsequently, probiotics have been also proposed in dentistry as adjuncts in tooth decay, gingivitis, periodontitis, and candidiasis management [4]. Although probiotics’ mechanism of action in the oral cavity is still ignored, they seem to directly act on the breakdown of dental plaque and indirectly modulate the host’s immune response [24,25].

The most used probiotics for diseases affecting the oral cavity comprise Bifidobacteria and Lactobacilli, and several posology and administration protocols have been tested and are still under study for oral mucosal, dental, and periodontal/peri-implant tissue dysbiotic conditions in preventive and therapeutic perspectives [24,25]. 

Therefore, the present study aimed to review, considering the periodontal microbiome composition around teeth and dental implants both in healthy and pathological conditions, the putative favorable effects of probiotics on gingivitis, periodontitis, and peri-implantitis. The secondary aim of the present narrative review was to synthesize the supporting evidence and proposed protocols for probiotics use as adjuncts in periodontitis and peri-implantitis treatment and in the periodontal health management of orthodontic patients with fixed appliances.

## 2. Periodontal Microbiota

The oral cavity is characterized by several microenvironments, including periodontal pockets, tooth surfaces, cheeks, and tongue [26]. In healthy conditions, the temperature of the oral cavity is 37 °C, and the pH of the saliva ranges between 6.5 and 7.5, ideal conditions for the growth of numerous species of microbiota [27].

Microbiota refers to the set of archaea, fungi, protists, algae, and, above all, bacteria and their interactions, defined by some authors as the “theater of activity”. In detail, bacteria present proteins or peptides, lipids, polysaccharides, and nucleic acids as structural components, as well as metabolites, such as signaling molecules, toxins, and organic molecules [26,27]. 

### 2.1. Periodontal Microbiota in Healthy Conditions

*Aggregatibacter actinomycetemcomitans*, *Porphyromonas gingivalis*, *Prevotella intermedia*, *Capnocytophaga* spp., *Veillonella atypica*, and *Selenomonas* spp. are the main components of the tongue microbial flora [26]. Those bacteria form a multispecies organized community in the gingival crevicular areas, known as periodontal microbiota or gingival biofilm [28].

#### 2.1.1. Periodontal Microbiota around Natural Teeth in Healthy Conditions

The microbial communities developing on the teeth root surfaces and drawing nourishment from the gingival crevicular fluid, which is an exudate that flows into the gingival sulcus from the adjacent gingival tissues and is able to survive even the anaerobic conditions that can be established in the subgingival environments [29].

From the first microscopic studies, some bacterial species, especially Gram-positive rod and cocci, appeared numerically dominant at the subgingival level in healthy periodontal conditions. In detail, Actinomyces naeslundii was the most abundant subgingival species in healthy conditions [30,31]. 

However, other *Actinomyces* spp. were also present, including *A. meyeri* and *A. odontolyticus*, which have a high coping capacity and aggregate with other colonizing bacteria such as Streptococcus [29]. *Streptococcus sanguinis*, *S. intermedius*, *S. gordonii*, *S. oralis*, *Peptostreptococcus micros*, and *Gemella morbillorum*, among the Gram-positive species, and *Capnocytophaga ochracea*, *C. gingivalis*, *Veillonella parvula*, and *V. atypica*, among the Gram-negative ones, were also commonly found in the subgingival biofilm. *Fusobacterium nucleatum*, a Gram-negative filamentous, was the second most frequently detected species in healthy biofilm [29].

Subsequent studies based on 16S rRNA gene sequencing pointed out that *Actinomyces* spp. and streptococci of the Mitis group were the most abundant species, along with *Capnocytophaga* spp., *F. nucleatum*, and *V. parvula*. In addition, Gram-positive species have also been identified such as *Rothia aeria*, *R. dentocariosa*, *Corynebacterium durum*, and *C. matruchotti* [29,30,31].

#### 2.1.2. Periodontal Microbiota around Dental Implants in Healthy Conditions

Some authors highlighted that the normal microbiota of healthy dental implants is qualitatively similar to the subgingival microbiota with an equal composition of Gram-positive rods and cocci [32]. 

Conversely, other authors have described substantial differences between periodontal and peri-implant biofilm, potentially attributable to different mechanisms underlying biofilm formation on teeth compared to titanium surfaces. In this regard, the possible release of degradation products from dental implant metal surfaces in the peri-implant sulcus, which may cause stress on the microbiota, on the one side, and alter the local immune response to bacteria, on the other side, should also be considered [33].

### 2.2. Periodontal Microbiota in Pathological Conditions

#### 2.2.1. Periodontal Microbiota in Gingivitis and Periodontitis

After 2–3 weeks of abstention from oral hygiene, the accumulation of plaque causes a change in the species present at the subgingival level with an increase in Gram-negative bacteria. A decrease in *R. dentocariosa*, Propionibacterium from *Stenotrophomonas maltophila*, and an increase in Prevotella and Selenomonas were reported among the most critical subgingival microbiota changes, with Prevotella and Selenomonas representing the most strongly associated with both the increase of crevicular Interleukin (IL)-1a, IL-1b, and lactoferrin and the clinical signs of gingivitis [29,34,35,36]. 

Unlike gingivitis and mucositis, periodontitis and peri-implantitis were associated with considerable changes in subgingival bacterial species composition [1,35,36].

Indeed, in periodontitis, an abundance of the main suspected pathogenic bacteria, comprising *Porphyromonas gingivalis*, *Treponema denticola*, and *Tannarella forsythia*, which constitutes the so-called red complex triad, was frequently detected [37].

A higher microbial load from the so-called orange complex triad, composed of *Prevotella intermedia*, *Fusobacterium nucleatum*, and *Parvimonas micra*, as well as from *Aggragatibacter actinomycetemcomitans*, *Bacteroides forsythus*, *Campylobacter rectus*, *Firmicutes phylum*, *Eikenella corrodens*, *Filifactor alocis*, *Peptoanaerobacter stomatitis*, *Methanobrevibacter oralis*, archeon phylotype Thermoplasmata, Candida Albicans, human cytomegalovirus, and Epstein–Barr Virus was also noticed [36,37,38,39]. These microorganisms colonize periodontal subgingival sites, eluding host immune defense and directly or indirectly determining tissue damage and disease progression through their virulence factors [40,41]. Concomitantly, *Actinomyces* spp., *Rothia* spp., and *S. Sanguinis*, more abundant in periodontally healthy conditions, were found to be decreased in periodontitis [41].

In addition, a substantial modification in the tongue microbiota was found in gingivitis and periodontitis. Indeed, the dorsum of the tongue can be considered a reservoir for periodontal bacteria, thus contributing to the recolonization of treated subgingival and interdental sites. As a counterpart, the microbial composition of the tongue seems to be influenced by the interdental microbiome, which is characterized by the highest abundance and diversity of Fusobacteria, with *F. periodonticum* being the dominant species. Accordingly, tongue microbiota differed significantly in pathological compared to healthy conditions, harboring increased colonies of *F. nucleatum* ssp. polymorphum and *F. nucleatum* ssp. vincentii, indirectly reflecting the role of the interdental microbiome in tongue as well as subgingival biofilm composition [35].

#### 2.2.2. Periodontal Microbiota in Peri-Implantitis

The precise microbiome composition associated with peri-implant tissues’ unhealthy conditions was difficult to delineate due to several interplaying factors [42]. Consequently, no specific microbial species have been identified as harvesting exclusively or predominantly in dental implant sites with peri-implantitis compared to healthy ones. 

However, the peri-implantitis microbiome was reported to be characterized by high microbial diversity, consisting of aerobic Gram-positive, Gram-negative anaerobic, and pathogenic spindle-shaped rods. A higher prevalence of *T. denticola*, *P. intermedia*, *C. rectus*, and *Staphylococcus warneri*, as well as of *Bacteroidetes* spp., *Actinomyces* spp., *Campylobacter* spp., *Peptococcus* spp., *Streptococcus* spp., and *Butyrivibrio* spp., was found in peri-implantitis [43]. 

In addition, some studies have proposed that microbial flora in peri-implantitis biofilm may have a broader spectrum than periodontitis, and higher counts of human cytomegalovirus and Epstein–Barr Virus were detected in peri-implantitis biofilms [44]. 

Interestingly, a recent study analyzing periodontal and peri-implant pocket samples from the same patient found higher concentrations of *F. nucleatum*, *T. forsythia*, *F. necrophorum*, *P. micra*, and *C. rectus* in the microbiological composition in peri-implant sites. A higher prevalence of *P. intermedia*, *T. denticola*, *C. rectus*, and *Staphylococcus warneri* was found at inflamed sites comparing inflamed peri-implant sites with healthy ones [45] and of *P. nigrescens*, along with a lower concentration of *Peptostreptococcaceae* spp., in peri-implantitis versus periodontitis specimens [44].

## 3. Probiotics

The term probiotic was coined by Lilly and Stillwell in 1965 [40] from a combination of the Latin preposition pro (“in favor of”) and the Greek adjective βιωτικός (“biotic”) containing the noun βίος (“life”), so it means “in favor of life.” Eli Metchnikoff first hypothesized the protective role of live bacteria and evaluated the beneficial effects on human health of the extensive use of fermented milk. Subsequently, WHO defined probiotics as all “those living microorganisms that, when administered in adequate amounts, exert a positive effect on the host’s health by strengthening the gut ecosystem” [46].

Probiotics should meet specific requisites by definition, such as being safe for use in humans, i.e., not determining acquired or transmissible antibiotic resistance, thus listed among those bacterial species presumptively qualified as safe; being capable of resisting the high gastric, pancreatic, and bile juices’ acidity and of persisting and multiplying in the human intestine; and active and viable at the gastrointestinal level in proper quantities justifying beneficial effects observed in efficacy studies [47]. Based on these characteristics, Lactobacilli, including *Lactobacillus acidophilus*, casei, lactis, and bulgaricus, and Bifidobacteria, comprising Streptococcus thermophilus and Bifidobacterium bifidum, are considered probiotic microorganisms [48]. However, since the biological effects produced by probiotics are strain-specific, using a new bacterial strain, although belonging to species already in use, requires a reevaluation of safety and efficacy.

### 3.1. Probiotics Functions

As a whole, the class of probiotics performs many functions in individuals’ good health status maintenance [49]. Among them, the most renowned include the administration of Lactobacilli, which can convert lactose to lactic acid through the so-called lactic fermentation, to aid individuals intolerant to lactose in digestion [50]. This beneficial effect is secondary to the release by these bacteria of the galactosidase enzyme, breaking down lactose into glucose and galactose, which are more digestible components. In addition, it is widely accepted that probiotics effectively block diarrhea associated with antibiotics [51]. 

Moreover, probiotics are also considered helpful in treating infections in adults. In detail, the addition of Lactobacilli to the standard therapy prescribed to treat gastritis sustained by Helicobacter pylori has been found to reduce the frequency of stomatitis and constipation related to its presence in the stomach, despite not modifying bacterium eradication rate [52]. 

Furthermore, probiotic administration is considered an adjuvant therapeutic strategy in several gastrointestinal diseases, such as irritable bowel syndrome [53], and obesity [54], which are etiologically related to the alteration of the microbiota. 

An antimutagenic effect of some Lactobacilli (e.g., *Lactobacillus bulgaricus*) has also been proposed and likely linked to their binding to heterocyclic amines, which are produced during the cooking process of food by the carcinogens contained in meat. Evidence of probiotics as adjuvants in colorectal cancer treatment has also been proposed [55].

### 3.2. Probiotics Mechanisms of Action

Although the evidence clearly and definitively highlighting probiotics mechanisms of actions in human hosts is still lacking, advances in genome sequencing, microbiota analysis, and real-time in vivo sampling should help to acquire new data in the coming years [56,57]. 

Probiotics enzymes such as β-galactosidase and bile salt hydrolase [58,59] were found able to improve lactose digestion and human blood lipid profiles, respectively, in turn providing sufficient evidence to authorize Streptococcus thermophilus and Lactobacillus bulgaricus addition as components of yogurt in order to alleviate symptoms of lactose poor digestion.

However, along with enzyme formation, probiotic microorganisms are thought to act through various means, including immune function modulation, organic acids, and antimicrobial compounds production, interaction with the host resident microbiota and cells, and improving intestinal barrier integrity.

In detail, some probiotics, through their capsules and surface structures [57], have been shown to increase natural-killer cells activity and phagocytosis, upregulate antibody secretion, and directly interact with dendritic cells [60,61,62], resulting in implemented immune defenses against pathogens and increased responses to vaccines. In addition, probiotic strains can also increase anti-inflammatory cytokines with implications for reducing colitis and colon cancer [60]. 

Lactobacillus and Bifidobacterium generate through carbohydrate metabolism lactic and acetic acid, lowering luminal pH and discouraging the growth of pathogens, thus contributing to anti-inflammatory mechanisms and in interorgans signaling, and are, therefore, considered essential for host health and well-being [63,64].

Probiotic strains can interact with the host microbiota by competing for nutrients, cross-feeding, antagonism, and supporting microbiota stability [65]. Moreover, probiotics interact with the host cells through surface proteins, mucin-binding proteins, and pili, as well as through nonprotein components, such as exopolysaccharides, peptidoglycan, and lipoteichoic acid [56]. Such interactions at the intestinal level result in improved gut barrier integrity, which is crucial to the survival of the individual, allowing the absorption of nutrients and defending the body from the entry of unwanted, often harmful macromolecules [56]. 

Some probiotic strains have also been demonstrated to be capable of producing small molecules exerting local effects, including acetylcholine, oxytocin, norepinephrine, dopamine, serotonin, tryptamine, and gamma-aminobutyric acid [66,67], as well as adrenocorticotropic hormone and corticosterone, in rats [68]. 

## 4. Probiotics’ Effect in Periodontal and Peri-Implant Diseases

Probiotics are live microorganisms divided into eight main classes, seven represented by bacteria and one by yeast [21,23]. 

Several clinical studies have already demonstrated some probiotics’ efficacy in treating systemic and infectious diseases [8,23]. Indeed, probiotics are well known to positively affect the gut microbiota, reducing the duration of antibiotic-associated diarrhea [69]. In addition, considering that probiotics administration side effects appear to be minimal, with mild gastrointestinal side effects, such as gas, and severe adverse effects, still under study, have been infrequently noticed, research on probiotics is currently regarded as very topical.

An equilibrium between the periodontal and peri-implant microbiota and tissue host cells is maintained under healthy conditions. Such microbiota is the essential etiologic factor for both periodontal and peri-implant diseases. Therefore, since in pathological conditions it switches into dysbiotic, as demonstrated by several animals [70,71] and human models [72], a potential beneficial effect of probiotics on periodontal and peri-implant dysbiosis has been proposed [20,73,74]. 

Moreover, probiotics have been found able to modulate host immune-inflammatory response; thus, a potential beneficial effect on periodontal tissue destruction has been hypothesized [22,23].

### 4.1. Probiotics’ Effects on Gingivitis: Current Evidence

Several probiotics, including *Lactobacillus reuteri* (*L. reuteri*), *Bifidobacterium animalis*, and *Bacillus* sp., were evaluated for their clinical efficacy in gingivitis [75,76,77] and found to be effective in reducing periodontal inflammatory parameters, such as bleeding on probing, gingival index, and biofilm accumulation. In particular, a recent randomized controlled clinical trial involving 51 patients with 28 days of follow-up reported a significant reduction in plaque and bleeding scores with a daily administration of yogurt containing Bifidobacterium animalis compared to the placebo group [78]. 

Similarly, *L. salivarius* WB21 and *L. reuteri* determined a significant reduction of biofilm accumulation and inflammation in subjects with moderate/severe gingivitis and in salivary inflammatory markers in smokers. *L. reuteri* may act through different mechanisms comprising the secretion of two bacteriocins, reuterin and reutericyclin, capable of inhibiting pathogen growth, shown in vitro by negative mRNA upregulation, the adhesion to host tissues, consequently competing with pathogenic species and anti-inflammatory effects, with IL-8 secretion induced by TNF-a [79].

However, since Alkaya et al., using a combination of three probiotics, specifically *Bacillus subtilis*, pumulus, and megatherium, described no significant differences in bleeding on probing, gingival, and plaque indices at 8 weeks follow-up between the test and placebo groups, the potential favorable role of probiotics in gingivitis remains controversial [75,79].

### 4.2. Probiotics’ Effects on Periodontitis: Current Evidence

Periodontitis has been described as “a heterogeneous group of pathoses characterized by a predominance of specific infectious agents in the face of inadequate local host defenses” where there is a balance between protective and destructive immune responses [23,80]. Poor oral hygiene, facilitating bacteria accumulation within the biofilm, influence allogenic shifts in the microbial community, leading to the onset of periodontal inflammation, which triggers the destruction of periodontal connective tissue, ligament, and alveolar bone. As described above, Gram-negative anaerobic bacteria, principally *Aggregatibacter actinomycetemcomitans*, *P. gingivalis*, *Treponema denticola*, and *Tannerella forsythia*, are considered to be pathogenic to periodontal tissues. In detail, *A. actinomycetemcomitans* and *P. gingivalis* have been found to be associated with an increased MMP-2 production, thus being potentially responsible for extracellular matrix disintegration and tissue destruction in periodontitis [23,81]. 

In order to manage periodontitis, a strategic approach is proposed to replace common periodontal pathogens with commensal oral microbes, and subgingival applications of *S. mitis*, *S. sanguis*, and *S. salivarius* were found effective in delaying periodontal pathogens’ recolonization [82]. After a replacement therapy with the same probiotic microorganisms, improvements in bone level and density in dogs were also observed [83].

Several studies showed improvements in periodontal indices after the use of probiotics. In particular, Gudrianov reported a reduction in periodontal inflammation following the intake of Bifidumbacterin tablets [84]. Lactobacillus brevis administered to subjects suffering from chronic periodontitis improved bleeding on probing and gingival and plaque indices [23], likely preventing nitric oxide production, positively regulating, in turn, prostaglandin E2 and MMPs salivary levels [85]. Staab et al. underlined in a case-control study that the subjects who drank a probiotic milk drink with *L. casei* Shirota had a higher biofilm accumulation and a significant reduction in MMP-3, elastase activity, and myeloperoxidase activity, but an increased plaque index was registered in both groups [86]. Periodontal outcomes following the use of a chlorhexidine-based toothpaste vs. a toothpaste containing probiotics (including Bifidobacterium), a toothpaste containing probiotics (including Bifidobacterium), and a toothpaste containing probiotics (including Bifidobacterium) plus chewing gum containing the same probiotics were tested in subjects with stage II–III periodontitis in conjunction with mechanical periodontal treatment, and subjects were significantly improved after 3 months. However, the reduction in periodontal pathogens load was observed only at the 3- and 6-month follow-up and affected only the orange complex, not the red, which hypothetically could be further expected as an indirect effect of the reduced species of the orange complex. The authors concluded that these results support the hypothesis that probiotics may exert their beneficial effects on both the inflammatory pathogenesis of periodontitis and its microbial etiology [87].

However, further studies on probiotics’ mechanisms of action should ascertain their therapeutic use in humans. Hence, available data indicate that probiotics may positively affect periodontal pathogens and clinical periodontal parameters. However, further trials employing probiotic microorganisms characterized by beneficial periodontal effects already obtained by in vitro studies are advocated [23,79].

### 4.3. Probiotics’ Effect in Peri-Implantitis: Current Evidence

Peri-implantitis is a pathological clinical condition affecting the tissues that support dental implants and is characterized by progressive bone loss, increased probing depth, and bleeding on probing [15,16].

Many authors have analyzed the influence of probiotic effects in peri-implantitis, but almost all have focused on the effect of probiotics combined or not with nonsurgical periodontal therapy.

Although oral probiotics have been proposed to improve periodontal disease treatment outcomes, only a few studies investigating their effectiveness in treating peri-implant diseases have been conducted, besides the shared etiopathogenesis and therapies among the diseases [88].

Mulla Munaz et al. demonstrated in vitro the susceptibility of various pathogenic bacteria found in peri-implantitis to *Lactobacillus salivarius* at a concentration of 50 mg/mL, thus concluding that *L. salivarius* probiotic could be effective at that dose at counteracting the primary pathogens involved in peri-implantitis, such as *P. gingivalis*, *P. intermedia*, *S. aureus*, and *S. salivaris* [86].

## 5. Probiotics in Periodontitis Treatment

Subgingival biofilm removal is the main goal for treating periodontitis since periodontal bacteria are considered the initiating factors of the disease [12,13,16]. This goal is primarily addressed through nonsurgical periodontal treatment (scaling and root planing, SRP) for mechanical biofilm control [89]. Combined with mechanical procedures, antimicrobial agents can also be used to reduce pathogens’ microbial load and improve clinical outcomes, particularly in recurrent and refractory cases [35,36]. 

However, the antibiotic resistance phenomenon and the frequent recolonization of treated sites by periodontal pathogens and disease recurrences have led to new approaches for managing periodontitis, affecting millions of people worldwide. 

Among those proposed innovative approaches, as already discussed, probiotics have been administered during the last decades to manage a multitude of infectious diseases, including oral dysbiosis and infections and, consequently, periodontal diseases, eliminating disease-causing pathogens and promoting healthy flora development [89]. It has, therefore, been proposed that probiotics may, as an adjunct to SRP, promote clinical periodontal improvement and immunological benefits by indirectly regulating anti- and proinflammatory cytokines production and periodontal biofilm formation and maturation [24,25].

### 5.1. Probiotics as Adjuncts to Periodontal Treatment: Measures of Biological and Clinical Outcomes

Although recently proposed, probiotics administration in periodontitis treatment seems promising. Future research is needed to clarify probiotics’ clinical and biological efficacy. Indeed, while the clinical efficacy, which is measured through periodontal clinical parameters, indirectly and potentially late phenotypically expresses biofilm control and inflammation reduction, the biological assessment may provide early results on probiotic treatment effectiveness.

In detail, the clinical periodontal parameters generally assessed are at least the clinical attachment level (CAL) values, measured as the distance between the cementoenamel junction and the periodontal pocket bottom in mm [90], and the probing pocket depth (PPD) values, the distance between the free gingival margin and the periodontal pocket bottom in mm [90], overall evaluating tissue destruction, and the bleeding on probing (BOP) dichotomic values, evaluating tissue inflammation [91]. 

From a biological point of view, it is important to understand if the treatment has acted by modifying the microflora. Different methods were used to assess this. In particular, the DNA–DNA checkerboard is considered an accurate, inexpensive, and quick method of assessing microbial biofilm composition [92]. With this approach, the health and disease-associated microflora in the supragingival and subgingival biofilm was assessed before and after periodontal treatment, and it was concluded that there was a decrease [93] in periodontal pathogenic bacteria of the red and orange complex [94]. However, other methods have been used, such as the Microbial shift multiplex PCR procedure, identifying bacteria in gingival biofilm samples [43,93,94]. Other techniques used for this purpose include, beyond the analysis of terminal restriction fragment length polymorphism, real-time PCR [94]. Indeed, real-time PCR and microarrays revealed a downregulation in inflammatory genes of periodontitis subjects after therapy. Therefore, such a technique may aid in revealing host genes crucially involved in the onset and development of periodontitis and in analyzing treatment efficacy [93], especially considering that host inflammation resolution is also essential in periodontitis treatment [95]. 

### 5.2. Probiotics as Adjuncts to Periodontal Treatment: Reported Biological and Clinical Outcomes

It has been described that streptococcal species applied to the teeth of dogs as an adjunct therapy after root planing resulted in a delay in periodontal pathogens recolonization and a decrease in tissue inflammation [82]. Human studies have also shown a reduction of periodontal pathogens after rinsing with mouthwash with added Bacillus subtilis and taking tablets containing *Lactobacillus salivarius* [96]. Some studies also showed that the administration of *L. reuteri* decreased the levels of proinflammatory cytokines, including IL-1B, TNF-α, and IL-8 levels [97]. In addition, Ince et al. also proposed that *L. reuteri* may aid in modulating the host’s microbiota, thus partially explaining probiotics’ beneficial effects on periodontitis treatment [97]. Accordingly, *L. reuteri* was found to be able to exert an anti-inflammatory effect against periodontal pathogens, such as *Aggregibacter actinomycetemcomitans*, *Prevotella intermedia*, *Porphyromonas gingivalis*, and *Fusobacterium nucleatum*. Indeed, Iniesta et al. revealed that *P. gingivalis* load was significantly decreased after the administration of *L. reuteri* [98]. Similarly, Haukioja et al. detected a reduced number of periodontopathogens binding sites in gingival biofilm consequent to the coaggregation of *F. nucleatum* and Bifidobacterium, indirectly pointing out the beneficial impact on periodontitis prevention and treatment of periodontal pathogens modulation [99]. 

However, from a clinical perspective, Iniesta et al. [98], although reporting that the adjunctive use of *L. reuteri* reduced the number of periodontal pathogens in the subgingival microbiota, did not observe any clinical improvements. On the contrary, Laleman et al. [100] found that the use of *L. reuteri* as an adjunct to mechanical treatment improved clinical periodontal parameters without impacting periodontal pathogens. Along with *L. reuteri*, two other Lactobacillus species, i.e., *L. brevis* and *L. salivarius*, have also improved periodontal clinical parameters in periodontitis, and after the use of the three Lactobacillus probiotics, a reduction in probing pocket depth and clinical attachment loss was recorded; gingival and plaque indices and bleeding on probing also generally improved secondary to probiotics administration in most of the studies [88]. However, the same beneficial results could not be noticed after the administration of Streptococcus species and *L. rhamnosus* SP-1, thus supporting the hypothesis of possible probiotics’ species specificity in periodontitis management [88]. Accordingly, the meta-analysis from Daoyong et al. showed that adjunctive probiotics in nonsurgical mechanical periodontal treatment provided a significant clinical improvement in PPD, CAL, and BOP in the short term, which were stable for at least three months, compared with the control groups not under probiotics [101]. However, such beneficial effects were less favorable at a 6-month follow-up [101]. Reported findings (Table 1) were declared to need further validation, considering the high heterogeneity in types and forms of probiotics used, periodontitis severity, population characteristics, and periodontal parameters recorded.

**Table 1:** Probiotics in periodontitis treatment.

Authors, Year	Type of the Article	Periodontitis Treatment	Periodontal Outcomes Measured	Main Results (Statistically Significant)
Gruner et al., 2016 [102]	Systematic review and meta-analysis	-Probiotics -Placebo	PPD BoP PI GI	Current evidence is sufficient for recommending probiotics in gingivitis and periodontitis management Probiotics significantly reduced bleeding-on-probing and gingival index, but not plaque index
Vives-Soler et al., 2020 [103]	Systematic review	-Probiotics + nonsurgical treatments -Nonsurgical treatments + placebo	BoP PI PPD	Probiotics may provide supplementary clinical improvements to manual debridement in chronic periodontitis
Jayaramanet et al., 2016 [104]	Systematic review	-Probiotics + mechanical debridement -placebo + mechanical debridement	BoP PI PPD	Probiotics produce only short-term microbiologic and clinical benefits in periodontitis treatment
Matsubara et al., 2016 [88]	Systematic review	-Probiotics + mechanical debridement -mechanical debridement alone -mechanical debridement + placebo	PPD CAL BoP	Oral probiotics administration may be considered effective and safe adjunct to scaling and root planing
Canut-Del Gado et al., 2021 [105]	Systematic review	Probiotics + mechanical debridement -mechanical debridement + placebo	PPD BoP	Probiotics administration as adjuvants to periodontal treatment may aid in improving the clinical outcomes
Nga Ho et al., 2020 [106]	Systematic review	-Nonsurgical periodontal therapy + Probiotics -nonsurgical periodontal therapy alone	PPD CAL	Heterogenous evidence supports the long-term clinical benefit of probiotics as adjuncts to nonsurgical periodontal treatment
Ikram et al., 2018 [107]	Systematic review and meta-analysis	-Scaling and root planning in the treatment of chronic periodontitis alone -Scaling and root planning in the treatment of chronic periodontitis + probiotics	PPD CAL	Adjunctive probiotics could result in additional benefits in CAL gain
Ng Ethan et al., 2022 [108]	Systematic review and meta-analysis	-Probiotics + mechanical debridement -mechanical debridement + placebo	PPD	Adjunctive probiotics should be considered safe and could offer beneficial effects compared to a placebo
Yanine et al., 2013 [109]	Systematic review	-Placebo vs. probiotics		Insufficient evidence currently supports the benefits of systemically-delivered probiotics in subjects suffering from periodontitis
Gheisary, 2022 [110]	Systematic review and meta-analysis	-Prevention and treatment of periodontitis + probiotics -Prevention and treatment of periodontitis alone	PI GI PPD BoP	Probiotic administration improves clinical periodontal parameters

Abbreviations: Periodontal probing depth, PPD; BoP, bleeding on probing; clinical attachment loss, CAL; plaque index, PI; gingival index, GI.

Although multiple administration protocols of probiotics in suspensions, tablets, and lozenges, with different treatment duration (4 days–12 weeks) and posology (1–4 times per day), have been tested in periodontitis treatment [79,88,102,103,104,105,106,107,108,109,110], future studies should point out standardized protocols to yield broadly comparable data [88].

## 6. Probiotics in Peri-Implantitis Treatment

Very little evidence is currently available evaluating and supporting the rationale and the efficacy of probiotics as adjuncts in peri-implantitis treatment, which is anyway borrowed from periodontitis management aims and methods. 

It is noteworthy that Sargolzaei et al. [111] compared in a randomized double-blind study the effects of probiotic tablets with placebo ones on periodontal conditions of patients with peri-implant mucositis after nonsurgical periodontal treatment. The results obtained after 28 days of probiotics administration showed a statistically significant difference in BOP values between the probiotic and the placebo groups, whereas no differences were found in PD values. Other studies [74,112,113,114,115,116] reported severely lacking and contrasting results (Table 2). 

**Table 2:** Probiotics in peri-implantitis treatment.

Authors, Year	Type of the Article	Peri-Implantitis Treatment	Periodontal Outcomes Measured	Main Results (Statistically Significant)
Zhao et al., 2021 [112]	Systematic review and meta-analysis	-Probiotic therapy + mechanical debridement -Mechanical debridement alone -Mechanical debridement + placebo	PPD BoP PI	No differences between the groups
Sayardoust et al., 2022 [74]	Systematic review and meta-analysis	-Probiotics -Probiotics + nonsurgical treatments -Nonsurgical treatments	BoP GI PPD	No differences between the groups
Arbildo-Vega et al., 2021 [113]	Systematic review and meta-analysis	-Lactobacillus reuteri + mechanical debridement -Mechanical debridement alone	BoP PI PPD	PPD improvement has been observed in the group using Lactobacillus reuteri
Gao et al., 2020 [114]	Systematic review and meta-analysis	-Lactobacillus + mechanical debridement (MD) -Mechanical debridement alone -Mechanical debridement + placebo	PPD PI BoP	Lactobacillus provided limited benefits in peri-implant mucositis for PD. No significant differences were found for PI and BOP
Silva et al., 2020 [115]	Systematic review	Effect of probiotics on peri-implant diseases	PPD BoP PI GI	No clinical effects of probiotics were observed
Pietri et al., 2020 [116]	Systematic review	Probiotic therapy among patients undergoing fixed orthodontic therapy	PI GI Halitosis	Many studies reported that probiotic therapy had a beneficial effect on PI, GI, and halitosis among patients undergoing fixed orthodontic therapy

Abbreviations: Periodontal probing depth, PPD; BoP, bleeding on probing; plaque index, PI; gingival index, GI.

Consequently, very insufficient data are currently available to confirm the effective use of probiotics in managing peri-implantitis. Further studies are needed to understand the optimal type, dose, and duration of probiotics administration to obtain the greatest benefit in peri-implantitis treatment.

## 7. Probiotics in Orthodontic Treatment

Patients undergoing fixed orthodontic therapies frequently experience difficulties maintaining good oral hygiene during treatment [117,118,119,120]. Indeed, fixed orthodontic appliances are recognized to facilitate biofilm accumulation on both teeth and appliance surfaces [119]. Therefore, along with common home care recommendations and practice, probiotics have also been proposed, not only to control cariogenic biofilm, thus reducing the risk of white spots and cavities associated with fixed orthodontic treatments, but also to properly manage gingival biofilm and, especially, periodontal pathogens.

Consequently, several studies [116,119,121,122,123] have pointed out that probiotics effectively reduced pathogenic bacteria counts, specifically comprising *P. gingivalis*, Lactobacillus species, and *S. mutans*, in dental plaque samples and/or saliva. Accordingly, Shah et al. [119] also found clinical improvements in PI and GI values, although Benic et al. [118] reported opposite results. 

Therefore, it may be concluded that the studies investigating microbiological and clinical results following probiotics prescriptions in orthodontic patients have reported contrasting results. Such discordances may be due to the different methods, posology, and duration of probiotics prescriptions and to the heterogeneous biological and clinical measurement methods employed. Further studies are necessary to highlight the potential beneficial role of probiotics as adjuncts to home care in patients undergoing fixed orthodontic therapy.

## 8. Probiotics’ Effects on Periodontal Dysbiosis Reversal and Biofilm Control around Natural Teeth and Dental Implants

Probiotics have different functions, which include encouraging the development of microflora, counteracting pathogenic bacteria colonization through the decrease of luminal pH and the availability of the substrate to other bacterial populations, and the production of inhibitory compounds [23,73,74].

**Figure 1:**
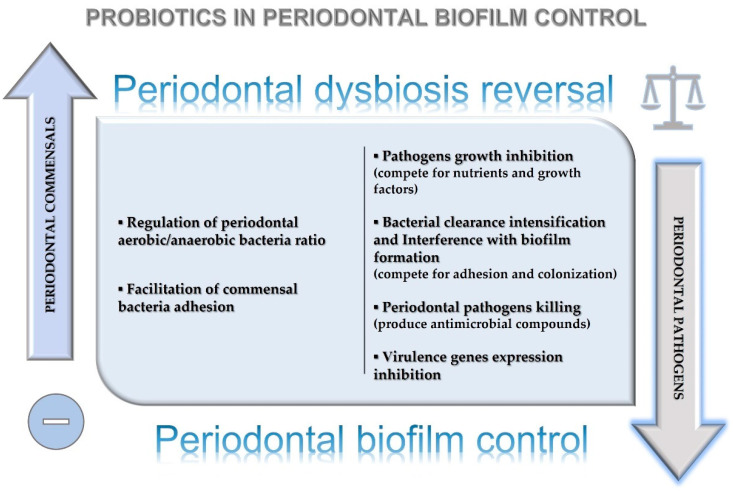
Putative probiotics’ effect on periodontal dysbiosis reversal and biofilm control in periodontal health management.

Putative probiotics’ effects on periodontal dysbiosis reversal and biofilm control in periodontal and peri-implant health management have been illustrated in Figure 1.

## 9. Probiotics’ Effects on Host Modulation in Periodontal and Peri-Implant Tissues

Probiotics increase the mucous membrane trophism and the epithelial barrier [124], stimulate the immune system, and modulate host-mediated inflammation [125]. 

In detail, immune response stimulation may be induced by probiotics by improving humoral immune responses, stimulating nonspecific host response to microbial pathogens, and favoring the mucosal immunological barrier. Furthermore, immune response stimulation seems to occur through the production of immunoglobulins (IgA), defensins, and cytokines and by decreasing the production of metalloproteinases (MMP) [23,125].

Probiotics, along with statins, bisphosphonates, NSAIDs, and proresolving modulators, are considered periodontal host modulatory agents. Host modulation aims at regulating the inflammatory tissue response, thereby interrupting the self-perpetuating vicious cycle and associated damage, which may help reverse the dysbiotic milieu, on the one hand, and induce anti-inflammation and tissue repair, on the other [126]. 

Specifically, probiotics are thought to inhibit Th17 lymphocytes, which are critically involved in the excessive release of IL-17 [127,128,129]. Indeed, under physiological conditions, Th17 lymphocytes are regularly maintained in periodontal tissues and mucosal surfaces of the skin, lung, and gastrointestinal tract, suggesting a protective role in the oral barrier, particularly against Gram-negative bacteria and fungi [127,128,129,130]. However, excessive IL-17 levels have been detected in gingival crevicular fluid and inflamed periodontal tissues. 

The inflammation enhanced by IL-17 is mainly driven by its combined functions with other proinflammatory mediators and the recruitment of neutrophils [127], which in turn stimulate the secretion of IL-17. IL-17-associated inflammation has also been found in psoriasis, rheumatoid arthritis, and periodontitis and likely represents one of the pathogenic links between these diseases and periodontitis [131,132].

Therefore, probiotics could downregulate the IL-17 inflammatory pathway, indirectly reducing neutrophil recruitment, proinflammatory mediators’ levels, connective tissue destruction, and bone resorption in inflamed periodontal sites. 

However, host modulation should be further investigated in the comprehensive management [133] of periodontitis and peri-implantitis for its potential innovative clinical applications since it may represent an additional complementary strategy to mechanical periodontal treatment, especially considering that most of the other host modulatory agents are drugs and thus not without potential side effects.

**Figure 2:**
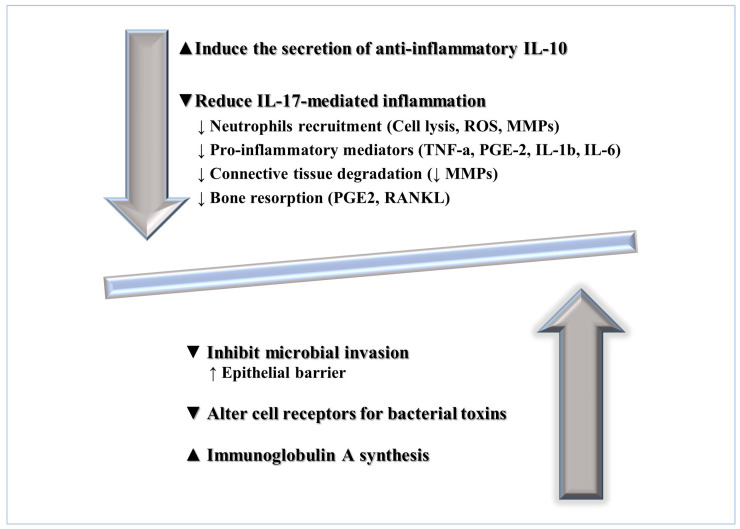
Putative probiotics’ effects on host modulation in periodontal and peri-implant tissues. Abbreviations: IL-10, Interleukin-10; ROS, reactive oxygen species; MMPs, matrix metalloproteinases; TNF-a, tumor necrosis factor-alfa; PGE-2, Prostaglandin-2; RANKL, Receptor Activator of Nuclear Factor κ B Ligand.

Putative probiotics’ effects on host modulation in periodontal and peri-implant health management are illustrated in Figure 2.

## 10. Conclusions

Probiotics are living microorganisms that, given in certain quantities, confer health benefits on the host. They were first used for the treatment of vaginal and intestinal infective and inflammatory conditions and later also employed in dentistry as adjuncts in tooth decay, gingivitis, periodontitis, and candidiasis management. 

Thus, some authors have suggested the use of probiotics as adjuvants in periodontitis and peri-implantitis therapy based on the dysbiotic etiology of both diseases and on their potential role in host inflammation modulation. Although multiple administration protocols of probiotics in suspensions, tablets, and lozenges, with different treatment duration (4 days–12 weeks) and posology (1–4 times per day), have been tested in periodontitis, future studies are needed to point out standardized protocols providing the optimal type, dose, and duration of probiotics administration. In addition, very insufficient data were available to confirm the effective use of probiotics in managing peri-implantitis. 

Probiotics have also been proposed in periodontal health management strategies of orthodontic patients with fixed appliances. However, the potential beneficial role of probiotics as adjuncts to home care in patients undergoing fixed orthodontic therapy remains controversial due to the different methods, posology, and duration of probiotics prescriptions tested and the heterogeneous biological and clinical measurement methods employed. 

Therefore, further studies are needed to assess probiotics’ direct effect on periodontal dysbiosis reversal and biofilm control and indirect ones on host modulation, which may be particularly relevant in refractory and recurrent cases and/or at periodontal sites, validate their use in periodontal and peri-implant health management, and provide case-specific recommended protocols in healthy and pathological conditions.

## Data Availability

Data supporting reported results can be found on PubMed/MEDLINE, Scopus, Web of Science databases.
